# A Rapid Optical Clearing Protocol Using 2,2′-Thiodiethanol for Microscopic Observation of Fixed Mouse Brain

**DOI:** 10.1371/journal.pone.0116280

**Published:** 2015-01-29

**Authors:** Yuka Aoyagi, Ryosuke Kawakami, Hisayuki Osanai, Terumasa Hibi, Tomomi Nemoto

**Affiliations:** 1 Research Institute for Electronic Science, Hokkaido University, Sapporo, Hokkaido, Japan; 2 Graduate School of Information Science and Technology, Hokkaido University, Sapporo, Hokkaido, Japan; 3 Core Research for Evolutional Science and Technology (CREST), Japan Science and Technology Agency (JST), Kawaguchi, Saitama, Japan

## Abstract

Elucidation of neural circuit functions requires visualization of the fine structure of neurons in the inner regions of thick brain specimens. However, the tissue penetration depth of laser scanning microscopy is limited by light scattering and/or absorption by the tissue. Recently, several optical clearing reagents have been proposed for visualization in fixed specimens. However, they require complicated protocols or long treatment times. Here we report the effects of 2,2′-thiodiethanol (TDE) solutions as an optical clearing reagent for fixed mouse brains expressing a yellow fluorescent protein. Immersion of fixed brains in TDE solutions rapidly (within 30 min in the case of 400-µm-thick fixed brain slices) increased their transparency and enhanced the penetration depth in both confocal and two-photon microscopy. In addition, we succeeded in visualizing dendritic spines along single dendrites at deep positions in fixed thick brain slices. These results suggest that our proposed protocol using TDE solution is a rapid and useful method for optical clearing of fixed specimens expressing fluorescent proteins.

## Introduction

Elucidation of various biological functions requires visualization of fine cellular structures at large to small scales within tissues. Particularly, in the field of neuroscience, connectomics, the construction of connectional maps of neural circuits, has been investigated recently [1, 2]. For the comprehensive investigation of the connectivity of neurons within brains, a simple and efficient method for imaging wide and deep areas in the brain is required. In addition, visualization of detailed structures such as dendritic spines is expected to yield information about the underlying neural transmission. However, such studies have been restricted owing to the opacity of a fixed brain. The observable depth limit from the surface, the penetration depth, is approximately 1.4 mm in a living mouse brain [3, 4]. However, when the brain tissue is fixed to observe the detailed structures, the transparency is generally assumed to reduce. Therefore, the penetration depth is less than approximately 300 μm in fixed brain tissue, even in two-photon microscopy at the longer excitation wavelength of an unconventional laser, probably because of scattering and absorption of light by the specimen [5].

Recently, several optical clearing reagents have been reported for visualizing fixed whole brains [6–12]. However, these reagents require complicated protocols and/or treatment times of days to weeks to render fixed biological specimens transparent. Therefore, they often hinder the efficiency of biological experiments, so that it is difficult to provide a plenitude of biological insights. A glycol derivative, 2,2′-thiodiethanol (TDE), was previously reported as a mounting medium for adjusting the refractive index of specimens, and high-concentration solutions (95% or 97% TDE) reduced the opacity of fixed specimens of intestine or kidney stained with fluorescent dyes [13–15]. However, TDE has not been widely used as an optical clearing reagent, probably because high concentrations of TDE quench fluorescent proteins and have failed to identify the shape of cells [8, 13]. In this study, we demonstrated that immersion in lower concentrations of TDE rapidly rendered fixed specimens of brain tissue transparent without quenching the fluorescent protein. TDE-immersed brains showed enhanced penetration depths of approximately 2 mm in two-photon microscopy. We also succeeded in visualizing a hippocampal pyramidal neuron that extended three-dimensionally in TDE-immersed fixed brain slices and its dendritic spine shapes with confocal microscopy.

## Materials and Methods

### Ethics Statement

All protocols were approved by the Institutional Animal Care and Use Committee of National University Corporation Hokkaido University (Permit Number: 10-0119). All experiments were performed under general anesthesia, and all efforts were made to minimize suffering.

### Animals

**Figure 1:**
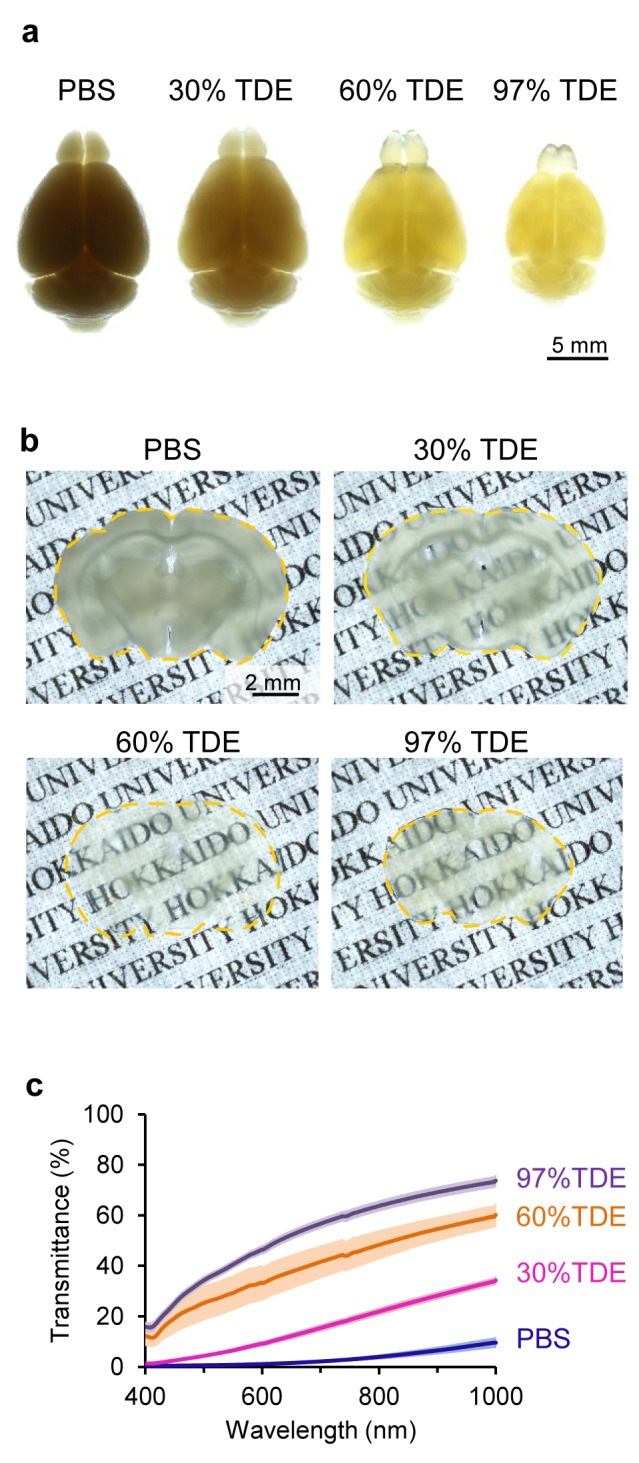
Fixed brain tissue clearing with TDE solutions. (a, b) Fixed adult mouse brains after treatment with different concentrations of TDE solutions. Whole brains (a) and brain slices (400 µm in thickness) (b) were immersed in each TDE solution for 2 days and 1 h, respectively. The photograms were taken under backlighting. (c) Transmission curves of fixed brain slices (400 µm in thickness, n = 3) after 2 h of immersion in each TDE solution. Data shown represent the average ± SEM.

Adult thy1-YFP-H transgenic mice expressing enhanced yellow fluorescent protein (EYFP) in subsets of hippocampal pyramidal neurons and layer V cortical pyramidal neurons [16], were used for fluorescent imaging and for preparing the fixed brain photograms shown in Figs. 1, S2, and S3. Adult C57BL/6 mice were used for the measurement of light transmittance.

### Preparation of fixed brain slices

For preparing the fixed brain photograms shown in Figs. 1, S2, and S3, the images of dendritic spines in a single hippocampal neuron, mice were anesthetized with pentobarbital sodium. They were transcardially perfused with phosphate-buffered saline (PBS) followed by 4% formaldehyde in PBS, and their brains were removed. Slices (400 or 500 µm in thickness) were prepared using a vibratome (7000smz, Campden, Instruments Ltd, UK). For the measurement of light transmittance, evaluation of the penetration depth in fixed hippocampal slices, and comparison of the combination of TDE and the objective lens, mice were anesthetized with isoflurane and their brains were removed quickly. Slices (400 µm in thickness) were cut using the vibratome in ice-cold artificial cerebrospinal fluid (containing 119 mM NaCl, 2.5 mM KCl, 1.0 mM NaH_2_PO_4_, 1.3 mM MgSO_4_, 2.5 mM CaCl_2_, 26 mM NaHCO_3_, and 10 mM glucose, bubbled with O_2_/CO_2_: 95%/5%) and incubated for 1 h at room temperature, followed by fixation with 4% formaldehyde in PBS.

### TDE treatment

TDE was purchased from Sigma-Aldrich (St. Louis, MO). Solutions of 0% (PBS only), 30%, 60%, and 97% TDE were prepared by mixing TDE, Milli-Q water, and PBS powder (Kohjin-Bio, Saitama, Japan). The refractive index of each TDE solution was measured with a pocket refractometer (PAL-RI, Atago, Tokyo, Japan) (S1 Fig.). The fixed slices and whole brains were immersed in the indicated concentrations of TDE solutions. Transparency was confirmed using a stereomicroscope (SZ61/SZ2-ILST, Olympus, Tokyo, Japan) and photos were taken using a camera (DP21, Olympus, Tokyo, Japan).

### Measurement of light transmittance

Light transmittance by fixed brain slices was recorded using a UV/visible/near infrared (NIR) spectrophotometer (U-2900, HITACHI, Tokyo, Japan). Wild-type mice were used to measure light transmittance. Thy1-YFP-H mice were not used for avoiding EYFP light excitation and emission. The hippocampal region of the fixed slices (400 µm in thickness) was superposed on a window in thick paper to measure light transmittance by only the hippocampal region.

### Imaging EYFP-expressing brain

All observations of EYFP-expressing brain for the measurement of the penetration depth were performed with an upright confocal and two-photon microscope system (A1R-MP and FN1, Nikon, Tokyo, Japan) equipped with a Ti: sapphire laser (MaiTai eHP DeepSee, SpectraPhysics, Santa Clara, CA). For observing brain slices, a 20× multi-immersion objective lens [Plan Fluor, numerical aperture (NA): 0.75, working distance (WD): 0.35 mm; Nikon, Tokyo, Japan] was used. The excitation wavelength was 488 nm (confocal) or 950 nm (two-photon). For imaging the whole brain, images were acquired with a 25× water-immersion objective lens (CFI Apo LWD, NA: 1.1, WD: 2 mm; Nikon, Tokyo, Japan). The excitation wavelength was 1010 nm. For observation of dendritic spines in the hippocampus, 60% TDE-immersed hippocampal slices were placed on a 0.06–0.08-mm-thick glass-bottom dish and covered with a paper wetted with 60% TDE. Images were acquired with the inverted confocal microscope system (A1R and Ti-E, Nikon, Tokyo, Japan). A 60× water-immersion objective lens (Plan Apo, NA: 1.2, WD: 0.27 mm; Nikon, Tokyo, Japan) or a 60× oil-immersion objective lens (Apo-TIRF, NA: 1.49, WD: 0.12 mm; Nikon, Tokyo, Japan) was used. The excitation wavelength was 514.5 nm. The emitted fluorescence signals within in the range of 525–555 nm, split with dichroic mirrors, were detected.

## Results

### TDE as an optical clearing reagent

Fixed adult whole brains were immersed in various concentrations of TDE in PBS (0%, 30%, 60%, and 97%) for 6 h to 7 days. Their opacity was reduced by treatment with 60% and 97% TDE for 2 days, and the fixed brains shrank in a concentration-dependent manner (Figs. 1a and S2). Next, fixed adult brain slices were immersed in the solutions. Immersion in 60% and 97% TDE for 1 h markedly and promptly rendered the fixed brains optically transparent (Fig. 1b). Although the slices shrank in a concentration-dependent manner (S3a Fig.), volume shrinkage was relatively suppressed by stepwise increases in TDE concentration (S3b Fig.). To characterize the effects of immersion in TDE, we measured light transmission spectra in a 400-µm-thick hippocampal region using a spectrophotometer. Light transmittance by the slices immersed in each TDE solution increased in the range from 400 to 1000 nm in a concentration-dependent manner (Fig. 1c). Five additional days of immersion in TDE changed the transmittance values negligibly (S4 Fig.). These results suggest that immersion in TDE solution facilitated observation by visible and NIR light.

### Imaging of hippocampal slices via laser scanning microscopy

**Figure 2:**
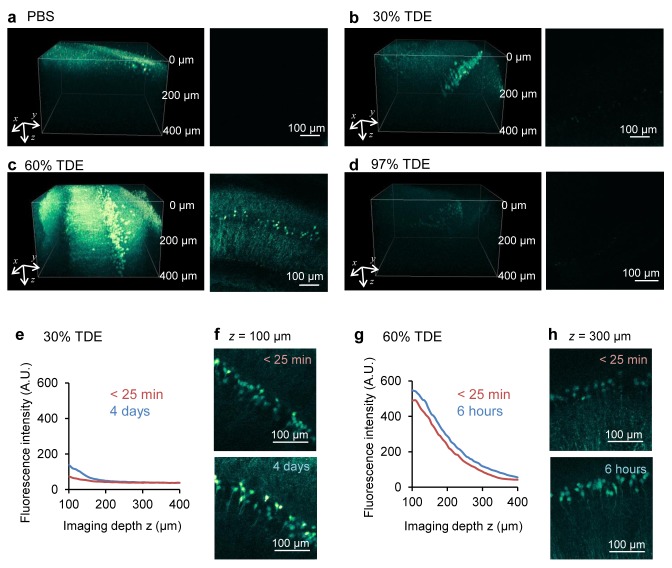
Enhancement of penetration depth on confocal microscopy. (a–d) Images of YFP-expressing neurons in the hippocampal slices of thy1-YFP-H mouse in PBS (a) and after 2 h of immersion in 30% TDE (b), 60% TDE (c), and 97% TDE (d) solution. Left, three-dimensional reconstructed images; right, *xy* images at a depth of 200 µm from the surface. (e, f) Fluorescence intensities immediately (within 25 min) and a long time (4 days) after immersion in 30% TDE solution. (e) Plot of the mean intensity of *xy* images against the depth from the surface; (f) Maximum projection images within 25 min and after 4 days of immersion in 30% TDE solution. (g, h) Fluorescence intensities immediately (within 25 min) and a few hours (6 h) after immersion in 60% TDE solutions. (g) Plot of the mean intensity of *xy* images against the depth from the surface; (h) Maximum projection images within 25 min and after 6 h of immersion in 60% TDE solution.

We investigated the relationship between TDE concentration and penetration depth in fixed hippocampal slices from thy1-YFP-H mice using confocal microscopy. Thy1-YFP-H mice express EYFP in subsets of hippocampal and layer V pyramidal neurons under control of the Thy1 promoter [16]. In the fixed brain slices immersed in only PBS, fluorescence signals were detected only in a region less than 50 µm deep from the surface (Fig. 2a). In contrast, 30% and 60% TDE enhanced the penetration depth in the fixed slices, as estimated from the non-saturated data in the deeper regions (Fig. 2b, c). The pyramidal neurons at a depth of 200 µm were visualized dimly or clearly after immersion in 30% or 60% TDE, respectively. In a 97% TDE-immersed slice, little signal was detected, similar to the results reported previously [8, 13] (Fig. 2d), probably owing to a side effect of TDE (S5 Fig.). We compared the depth dependence of the fluorescence intensity immediately after immersion in 30% or 60% TDE (within 25 min) and after a long time period. The fluorescence intensity immediately after immersion in 30% TDE was low through all depths. After immersion in 30% TDE for 4 days, the fluorescence intensity increased only in the region less than 160 µm deep (Fig. 2e, f). The fluorescence intensity immediately after immersion in 60% TDE was sufficiently higher at a depth of 100–300 µm. Additional immersion in 60% TDE for 6 h further increased the fluorescence intensity (Fig. 2g, h). These results suggest that immersion in 60% TDE immediately (within 25 min) enhanced the fluorescence intensity of the fixed brain slices.

**Figure 3:**
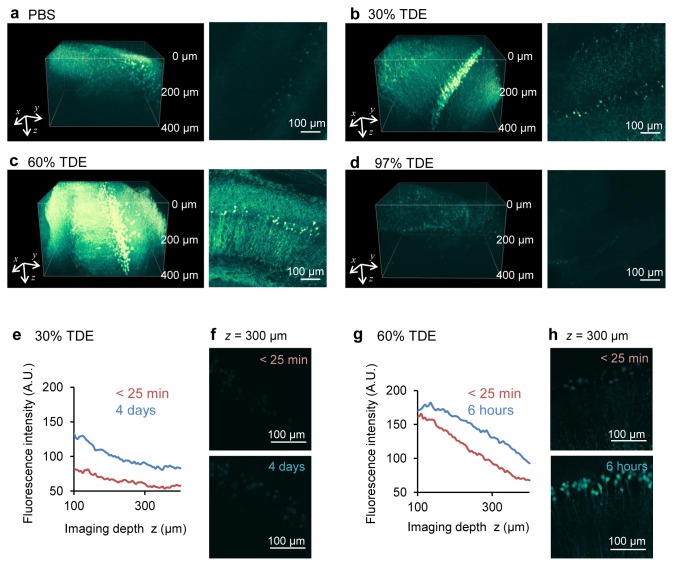
Enhancement of the penetration depth on two-photon microscopy. (a–d) Images of YFP-expressing neurons in the hippocampal slices of thy1-YFP-H mouse in PBS (a) and after 2 h of immersion in 30% TDE (b), 60% TDE (c), 97% TDE (d) solution. Left, three-dimensional reconstructed images; right, *xy* images at a depth of 200 µm from the slice surface. (e, f) Fluorescence intensities immediately (within 25 min) and a long time (4 days) after immersion in 30% TDE solution. (e) Plot of the mean intensity of *xy* images against the depth from the surface; (f) Maximum projection images within 25 min and after 4 days of immersion in 30% TDE solution. (g, h) Fluorescence intensities immediately (within 25 min) and a few hours (6 h) after immersion in 60% TDE solution. (g) Plot of the mean intensity of *xy* images against the depth from the surface; (h) Maximum projection images within 25 min and after 6 h of immersion in 60% TDE solution.

**Figure 4:**
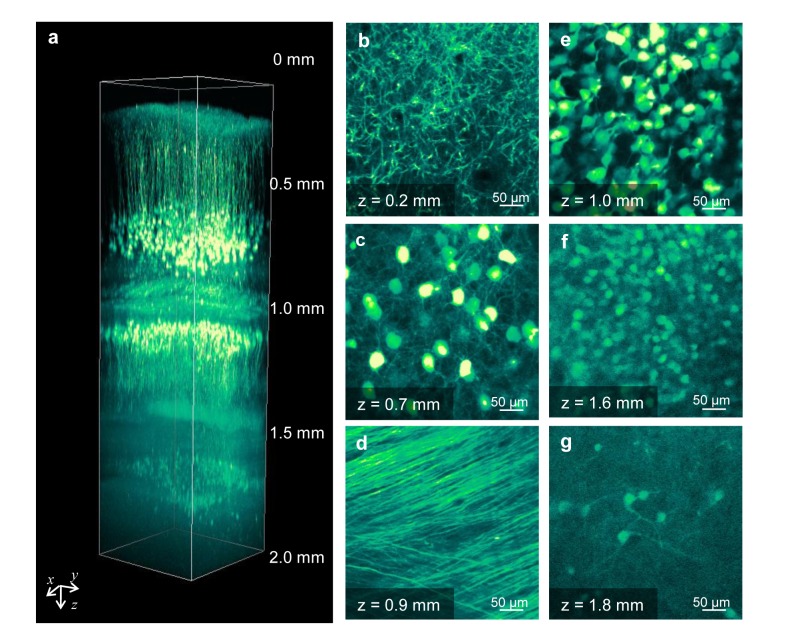
Two-photon deep imaging of a fixed whole brain immersed in 60% TDE. (a) Three-dimensional reconstructed image of a whole mouse brain after 2 days of immersion in 60% TDE. (b-g) *xy* images at different depths from the cerebral cortex to the lower portion of the hippocampus, including apical dendrites (b) and somata (c) of layer V neurons, white matter (d), hippocampal CA1 somata (e), upper blade (f), and lower blade (g) of the hippocampal dentate gyrus (DG).

Given that immersion in TDE increased transmittance, particularly in the NIR region, (Fig. 1c) of two-photon excitation laser light, we expected further enhancement of the penetration depth in two-photon microscopy. Immersion in 30% or 60% TDE clearly revealed the neurons in deeper positions of the fixed hippocampal slices (Fig. 3b, c). The intensity in the slice immersed in 60% TDE was higher than that in the slice immersed in 30% TDE. We compared the depth dependence of the fluorescence intensity in two-photon microscopy immediately after immersion in 30% or 60% TDE and after a long time period. In contrast to confocal microscopy, the fluorescence intensity increased through all depths following immersion in 30% TDE for 4 days (Fig. 3e, f). Although immersion in 60% TDE resulted in a high intensity within 25 min, similar to confocal microscopy, an additional 6 h of immersion led to an increase in intensity through all depths (Fig. 3g, h). Furthermore, we observed a fixed whole brain immersed in 60% TDE for 2 days using two-photon microscopy (Fig. 4). All layers of the cerebral cortex, the white matter, the CA1 region, and the dentate gyrus of the hippocampus were clearly visualized after immersion (Fig. 4b-g). These results showed that the 60% TDE solution was very useful and effective for the observation of the fixed specimens in two-photon microscopy.

### Visualization of dendritic spines in single hippocampal neurons

**Figure 5:**
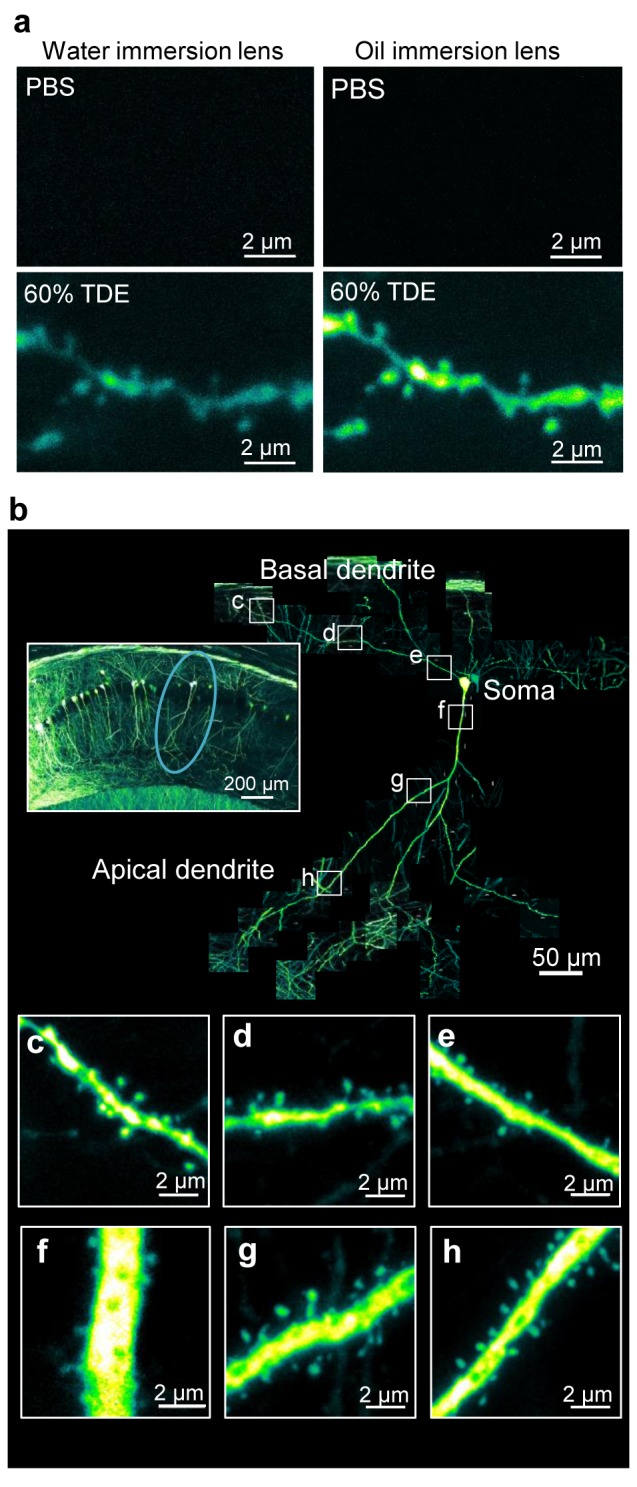
Images of dendritic spines along a single hippocampal neuron. (a) Combination of TDE treatment and water/oil-immersion objective lens with a high NA for imaging dendritic spine shapes in deep (100 µm) regions in a fixed brain slice. (b) Connected images of dendritic spines along single pyramidal neurons extending from a depth of 30 to 100 μm from the surface of the hippocampal slice. The left inset shows a low-magnification image of the hippocampus, and the circle shows the observed neuron. (c-h) Magnified images of the dendritic spine shapes on the basal dendrite (c-e) and the apical dendrite (f-h) along the neuron shown in (b). All images are maximum projection images.

Because TDE solutions have a higher refractive index than water (S1 Fig.), it was expected that use of an oil-immersion objective lens with a higher NA would result in brighter images. Confocal microscopic observations using TDE showed that the shapes of single dendritic spines in the fixed slices became clearer (Fig. 5a). Measuring the fluorescence intensities of dendritic spines showed that the combination of immersion in 60% TDE and an oil-immersion objective lens yielded images approximately 3 times brighter than those obtained with the water-immersion objective lens. This combination allowed identification of each dendritic spine along a single dendrite within an entire hippocampal neuron (Fig. 5b-h).

## Discussion

We evaluated a simple protocol using TDE solutions and showed that they rapidly rendered fixed brains optically transparent. Immersion in TDE for a short time increased the transmittance by fixed brain slices in a concentration-dependent manner (Fig. 1c), leading to enhancement of the penetration depth and an increase in the brightness of fluorescent cross-sectional images (Figs. 2, 3, and 4). Compared with other recently introduced clearing reagents such as CLARITY or CUBIC [10, 11], TDE solutions rendered the fixed adult whole brains less transparent; however, 60% TDE enhanced the penetration depth sufficiently to allow visualization of the structure of the hippocampus at approximately 2 mm from the surface. In other optical clearing protocols, a multi-step process (e.g., Sca*l*e, SeeDB, or CLARITY) [6, 8, 10] and long immersion time (e.g., Sca*l*e, CLARITY, or CUBIC) [6, 10, 11] are required. The principal advantages of our protocols are the single- or two-step process and the short immersion time. Our protocols using TDE solution can rapidly provide many results with less effort. Although *Clear^T^* has been reported to render embryos and neonatal brains transparent rapidly, the major component of its solution is toxic [9], whereas TDE is considerably harmless [13, 17] and thus easy to handle. It has been previously reported that TDE appears to be non-hazardous when exposed to skin and does not appear to pose an acute inhalation hazard [17]. Some clearing protocols with benzyl alcohol/benzyl benzoate (BABB) have been also reported to rapidly render tissues transparent [18–21]; however, these protocols require multi-step processes or cause significant shrinkage of the tissue. On the other hand, in our study, fixed brain slices were rendered transparent by a single-step protocol, except for stepwise increases in TDE concentrations to suppress the volume change (Figs. 5, S3b). The volume change in brain tissue is undesirable, because the volume of dendritic spines or the length of dendrites should be measured precisely for the elucidation of neural circuit functions. Furthermore, TDE is commercially available and inexpensive. Thus, the TDE clearing protocol may be readily performed in laboratories not skilled in imaging, even by inexperienced students.

The mechanism of optical clearing by TDE solutions is not clearly understood. After immersion in TDE, the fixed brain changed in appearance from white to largely transparent (Figs. 1b and S3), and no specific peaks or valleys in the transmission curve were evident (Fig. 1c). In a previous report, immersion in 80% TDE decreased the quantum yield of EGFP and mRFP to 71% and 85%, respectively [13]. Thus, we speculate that TDE reduces the difference in the refractive index between lipid bilayer membranes and the cytoplasm, resulting in a marked reduction in light scattering and transparency of a TDE-immersed specimen.

High-concentration TDE solutions (95% or 97%) have been reported to render biological tissues optically transparent; however, high-concentration solutions quench fluorescent proteins [13–15]. We found that low-concentration TDE solutions worked efficiently as a clearing reagent for fixed brains expressing a fluorescent protein (Figs. 2 and 3). Therefore, our rapid protocol could be applied to visualize the cells of interest by combination with genetic labeling methods. Time-lapse images of fixed slices immersed in 97% TDE from PBS showed that the fluorescence signals were initially highly detectable but rapidly decreased in intensity (S5 Fig.). After the fluorescence signals were almost eliminated by immersion in 97% TDE, we immersed the same slice again in PBS and subsequently into 60% TDE. This process recovered the fluorescence signals, and the structure of the neurons in the hippocampus appeared to be preserved (S6 Fig.). This result suggests that EYFP expressed in the cytoplasm remained inside the cells after treatment with 97% TDE, suggesting that TDE did not disrupt the cell membrane and did not cause EYFP leakage into the extracellular space. Hell’s group previously reported that the wavelengths of maximum fluorescence of EGFP and mRFP immersed in 80% TDE were shifted from 520 nm to 510 nm and from 606 nm to 608 nm, respectively, compared with the wavelengths after immersion in PBS [13]. In the present study, we observed EYFP signals in lower TDE concentrations. Therefore, it is difficult to directly compare our results with those reported by Hell’s group. We cannot exclude a possibility that a solvatochromic effect for EYFP decreased the fluorescence intensity on confocal microscopy following immersion in the TDE concentrations we used. However, such a slight shift of the wavelength would not be a major reason for the considerable decrease in fluorescence intensity, because we detected almost all the fluorescence in the wavelength range of 380–650 nm when we used two-photon microscopy where the two-photon excitation spectrum becomes wider. One possible reason for the intensity decrease is the change in the environment of the fluorophore of the fluorescent protein via dehydration [22, 23].

The simple and rapid TDE clearing protocol proposed here would be helpful for comprehensive studies of complex neural circuits or connectomics [1]. The enhancement of optical penetration deep into the whole brain by TDE treatment (Fig. 4) suggests that it is possible to investigate the interconnection of brain networks through multiple brain regions. The observation of fine shapes of dendritic spines in a single hippocampal neuron was achieved with the combination of 60% TDE treatment and a high-NA oil-immersion objective lens (Fig. 5). Dendritic spines are postsynaptic structures that receive synaptic exciting inputs from other neurons, and their morphologies have been linked with their functions, such as stability maintenance or synaptic transmission [24–26]. Visualizing the synaptic morphology could provide information on the connectional changes between normal and pathological brains, which have been reported to have synaptic disorders [27]. Immersion in TDE could also be combined with super-resolution microscopy to enhance its spatial resolution. However, the relatively short WD of a high-NA oil-immersion objective lens prevents observation at deep positions in a fixed tissue. Recently, the multi-immersion objective lens was designed with a high NA and long WD [6]. Development of an objective lens with a high NA and long WD would promote exploration of the neuronal network from large to submicron scales.

TDE solutions work as a simple and rapid optical clearing reagent for visualizing the fine structures of deep regions in fixed mouse brains. TDE solutions would be useful for visualizing other fixed organs expressing fluorescent proteins, because it was reported that a high concentration of TDE reduced the opacity of several fixed organs [14, 15]. Moreover, TDE solutions are compatible with immunohistochemistry [14, 15] and fluorescent proteins, suggesting that immersion in TDE would be compatible with the observation of specifically labeled structures. It is expected that the rapid optical clearing protocol using TDE proposed here will contribute to the accumulation of many biological insights and will help biological research advance at an accelerated pace.

## Supporting Information

S1 Fig Refractive index of TDE solutions. Plot of the refractive index against the concentration of TDE solution (n = 3). Data represent the average ± SEM. As previously reported [13], the refractive index increased in a concentration-dependent manner. The refractive index of the TDE solution is different from that of water (1.33) and standard immersion oil (1.52).
(TIF)

S2 Fig Time changes in fixed whole brains after immersion in each TDE solution. Photograms of fixed whole brains before and after immersion in 30%, 60%, and 97% TDE solutions for 6 h, 1 day, 5 days, and 7 days. The photograms at 2 days are shown in Fig. 1a. They were taken under backlighting.
(TIF)

S3 Fig Fixed brain slices before and after immersion in each TDE solution. (a) Photograms of fixed brain slices before and after immersion in each TDE solution for 3 h. (b) Photograms of the same fixed brain slice that was immersed in increasing concentrations of TDE (30%, 60%, 97%) every hour in a stepwise manner. Data represent the average ± SEM.
(TIF)

S4 Fig Time changes in light transmittance by hippocampal slices in TDE solutions. Plot of light transmittance by hippocampal slices (400 µm in thickness, n = 3) against treatment time. Transmittances at light wavelengths of 488, 527, and 950 nm are presented according to EYFP excitation and emission: 488 nm for confocal and 950 nm for two-photon microscopy excitation; 527 nm for detecting EYFP emission.
(TIF)

S5 Fig Time-lapse images of fixed brain slices after immersion in 97% TDE. (a) Images of fixed slices after immersion in 97% TDE. The images were observed using two-photon laser scanning microscopy. (b) Plot of the mean fluorescence intensity in the *xy* image at a depth of 300 µm from the surface. The fluorescence signal decreased over time after immersion in 97% TDE.
(TIF)

S6 Fig Recovery of the fluorescence signals eliminated after immersion in 97% TDE. (a) Image of a 97% TDE-treated fixed slice immediately after immersion in PBS. (b, c) Images of the same brain slice as shown in (a). The slice was incubated for 1 day in PBS (b), and then immersed in 60% TDE for 4 h (c). The fluorescence signals recovered and the structure of the hippocampal neurons appeared to be preserved. The images were observed using two-photon laser scanning microscopy.
(TIF)
