# Supplementary figures and images for: A Rapid Optical Clearing Protocol Using 2,2′-Thiodiethanol for Microscopic Observation of Fixed Mouse Brain
Source: PLoS One. 2015 Jan 29;10(1):e0116280. doi: 10.1371/journal.pone.0116280 (PMC4310605; doi:10.1371/journal.pone.0116280)

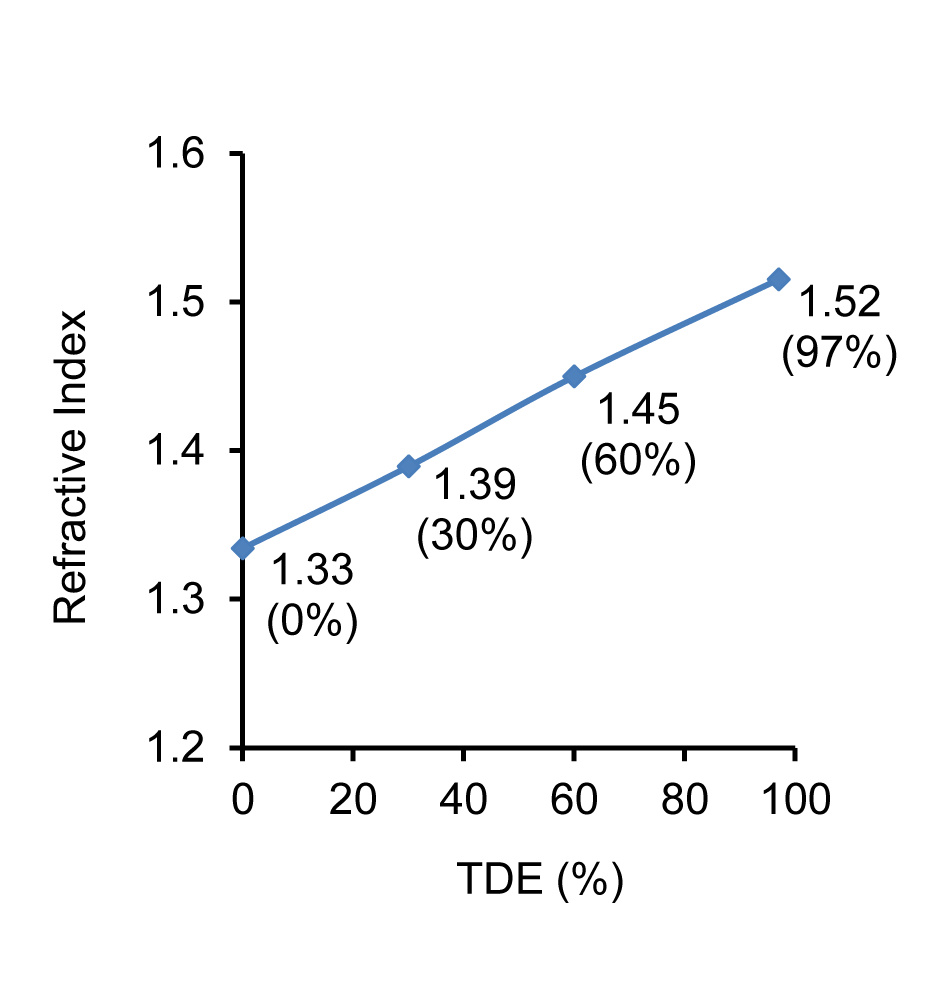

Supplement: S1 Fig — Plot of the refractive index against the concentration of TDE solution (n = 3). Data represent the average ± SEM. As previously reported [13], the refractive index increased in a concentration-dependent manner. The refractive index of the TDE solution is different from that of water (1.33) and standard immersion oil (1.52). (TIF) [file pone.0116280.s001.tif]

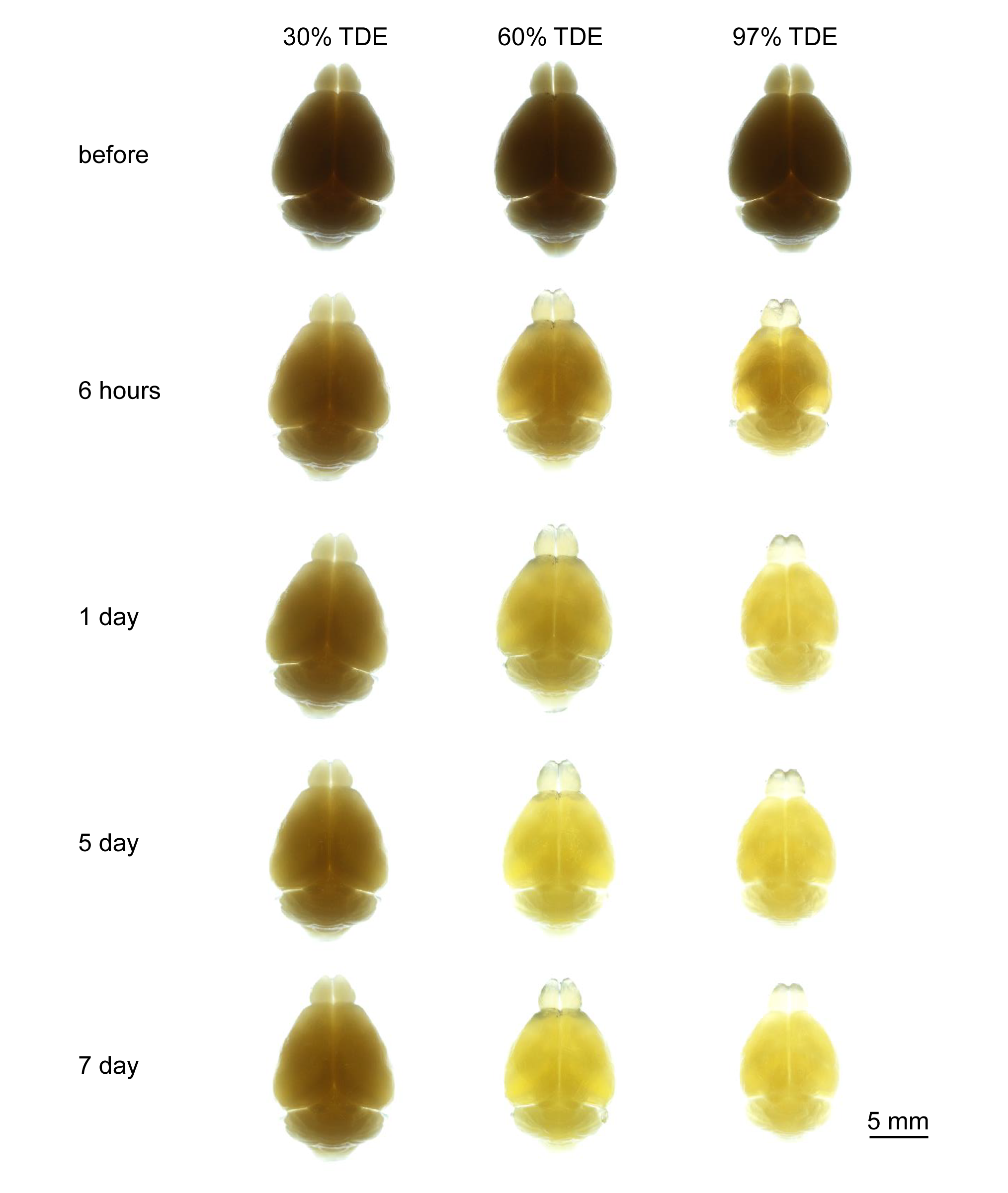

Supplement: S2 Fig — Photograms of fixed whole brains before and after immersion in 30%, 60%, and 97% TDE solutions for 6 h, 1 day, 5 days, and 7 days. The photograms at 2 days are shown in Fig. 1a. They were taken under backlighting. (TIF) [file pone.0116280.s002.tif]

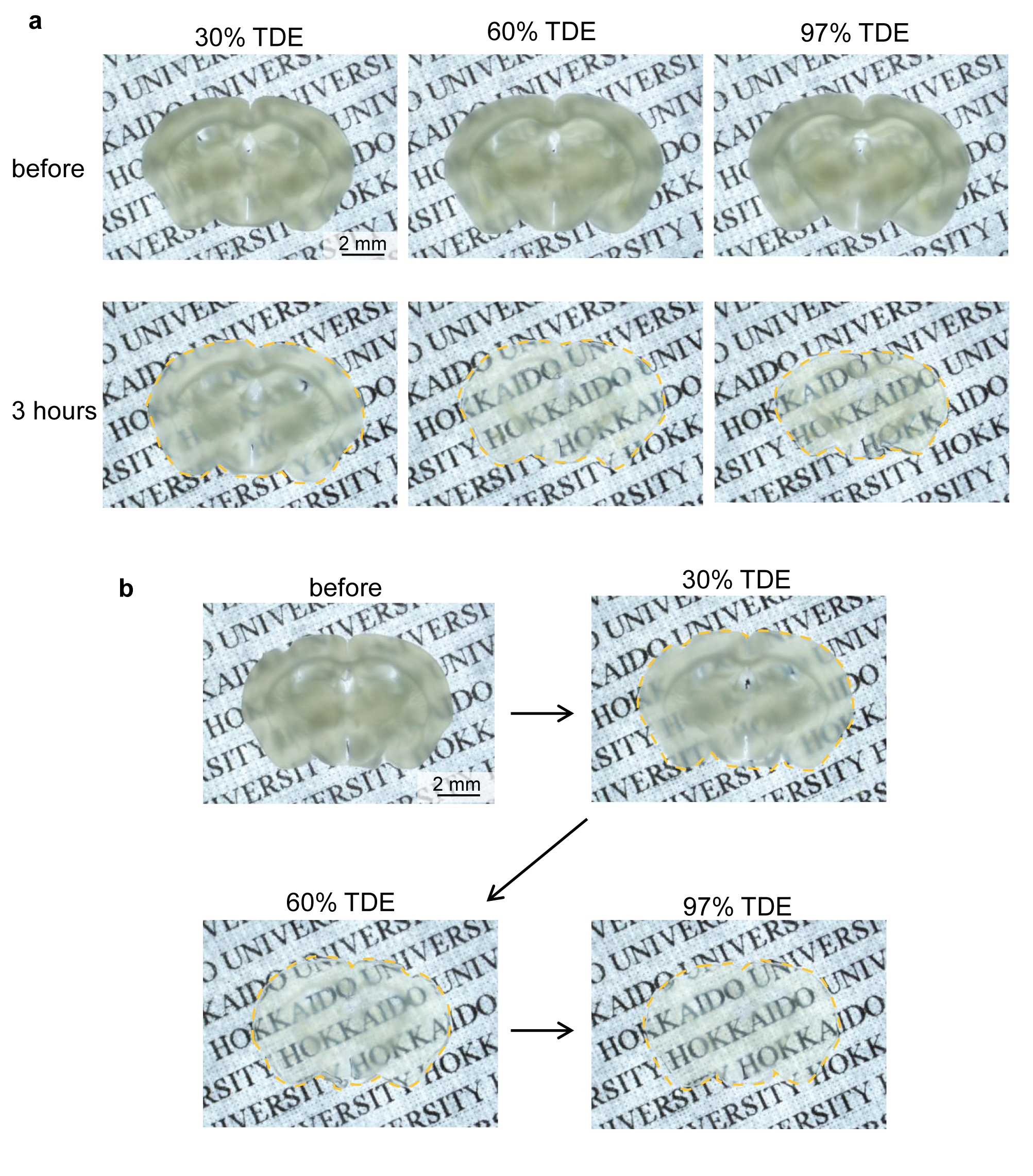

Supplement: S3 Fig — (a) Photograms of fixed brain slices before and after immersion in each TDE solution for 3 h. (b) Photograms of the same fixed brain slice that was immersed in increasing concentrations of TDE (30%, 60%, 97%) every hour in a stepwise manner. Data represent the average ± SEM. (TIF) [file pone.0116280.s003.tif]

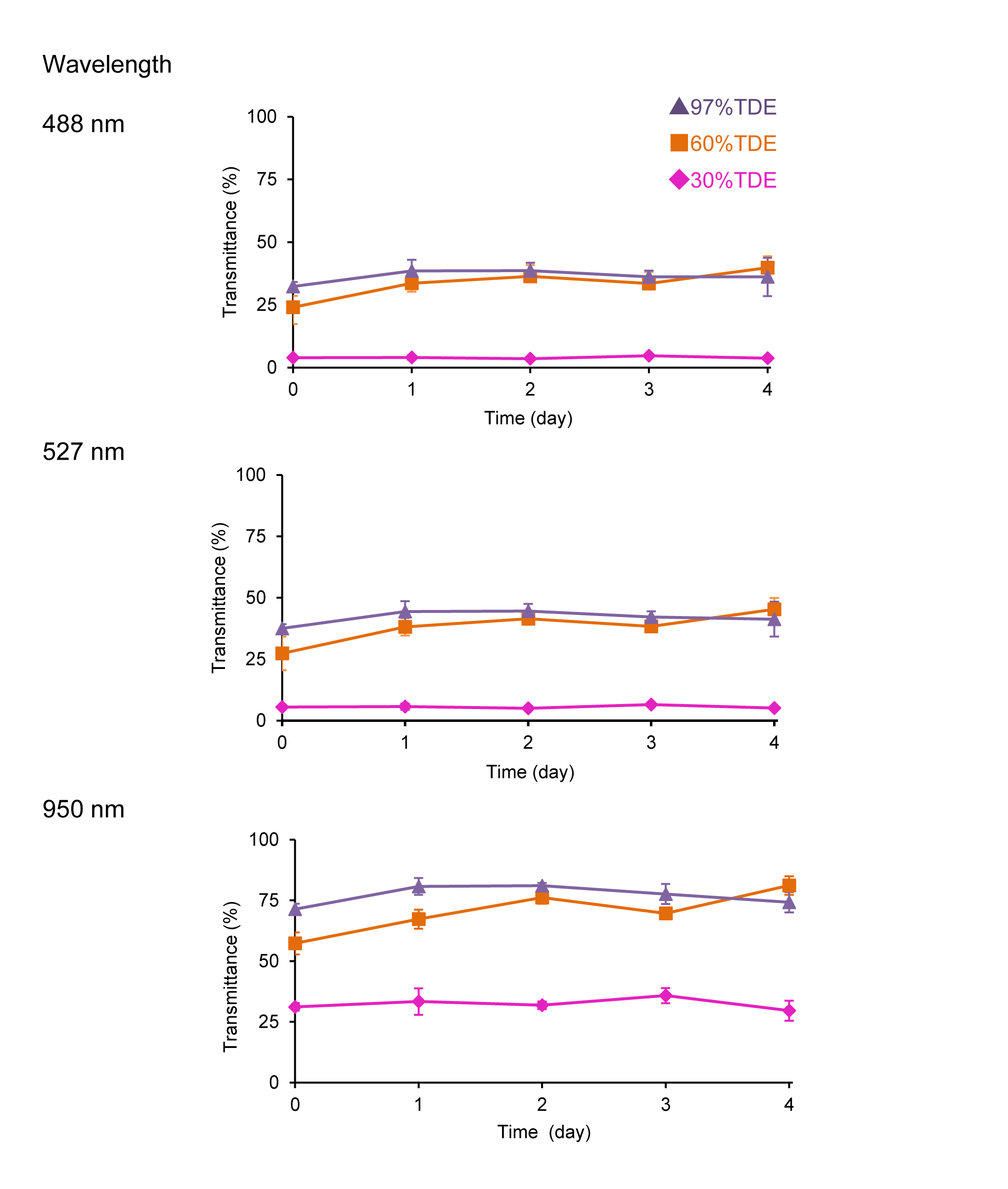

Supplement: S4 Fig — Plot of light transmittance by hippocampal slices (400 µm in thickness, n = 3) against treatment time. Transmittances at light wavelengths of 488, 527, and 950 nm are presented according to EYFP excitation and emission: 488 nm for confocal and 950 nm for two-photon microscopy excitation; 527 nm for detecting EYFP emission. (TIF) [file pone.0116280.s004.tif]

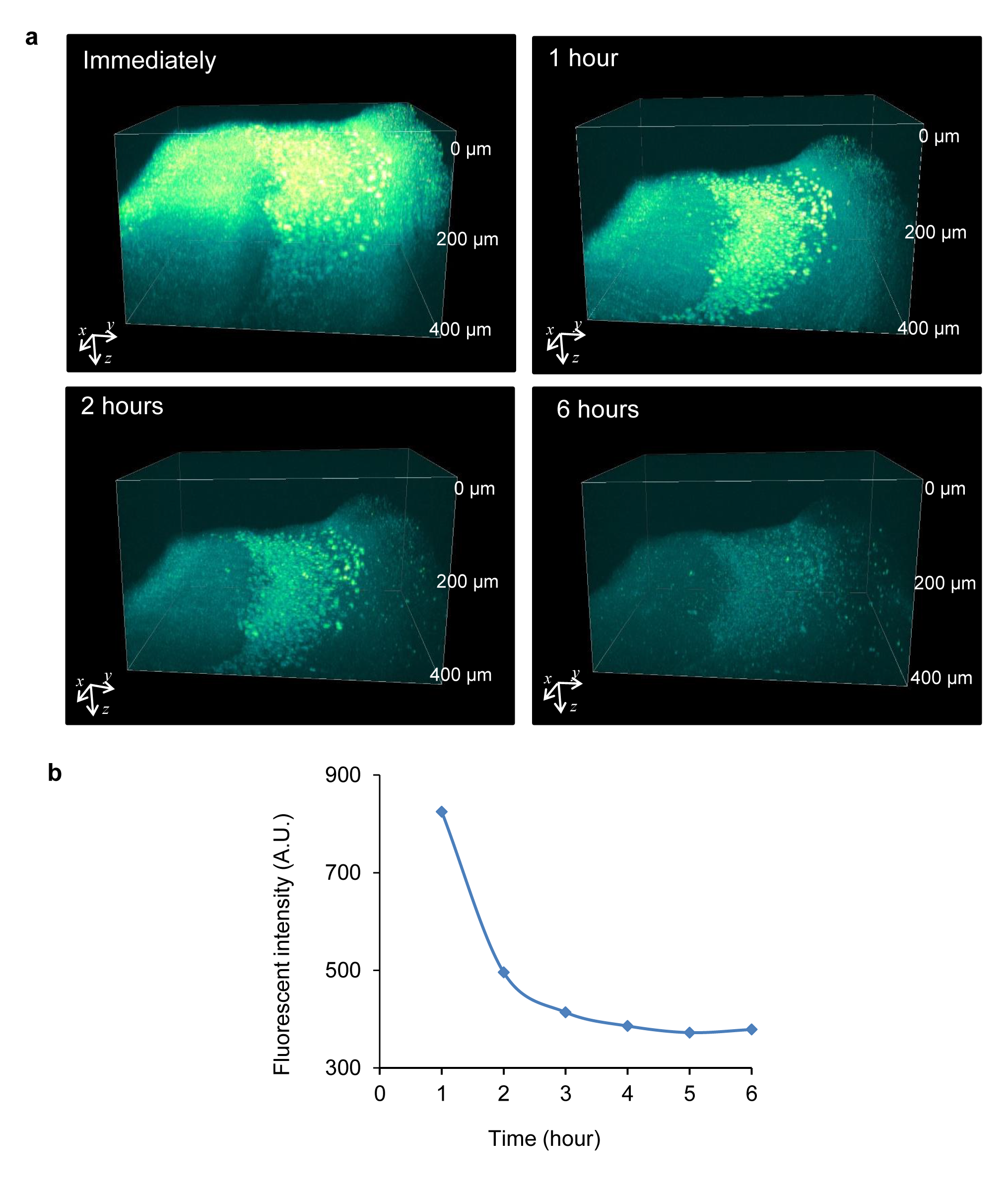

Supplement: S5 Fig — (a) Images of fixed slices after immersion in 97% TDE. The images were observed using two-photon laser scanning microscopy. (b) Plot of the mean fluorescence intensity in the xy image at a depth of 300 µm from the surface. The fluorescence signal decreased over time after immersion in 97% TDE. (TIF) [file pone.0116280.s005.tif]

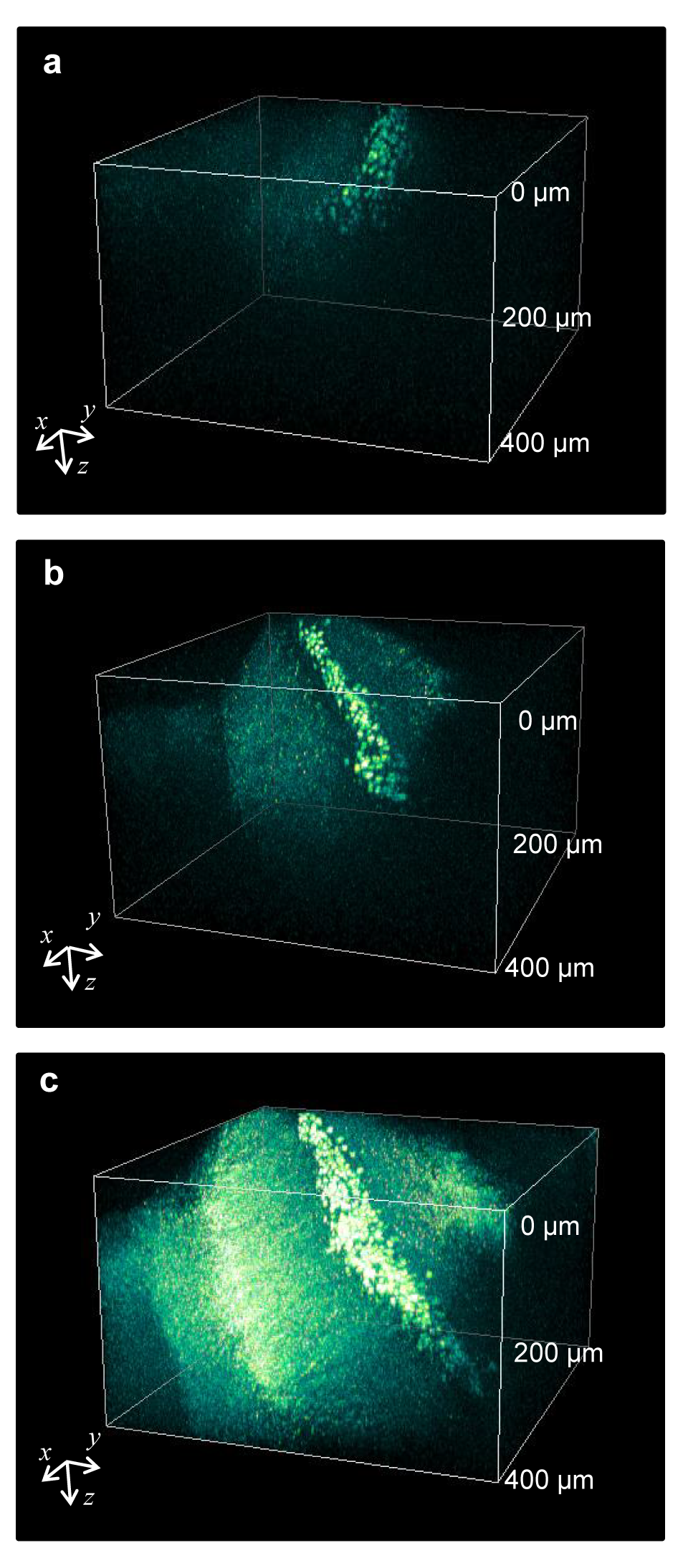

Supplement: S6 Fig — (a) Image of a 97% TDE-treated fixed slice immediately after immersion in PBS. (b, c) Images of the same brain slice as shown in (a). The slice was incubated for 1 day in PBS (b), and then immersed in 60% TDE for 4 h (c). The fluorescence signals recovered and the structure of the hippocampal neurons appeared to be preserved. The images were observed using two-photon laser scanning microscopy. (TIF) [file pone.0116280.s006.tif]
